# Spatiotemporal gait characteristics and ankle kinematics of backward walking in people with chronic ankle instability

**DOI:** 10.1038/s41598-020-68385-5

**Published:** 2020-07-13

**Authors:** Tharani Balasukumaran, Uri Gottlieb, Shmuel Springer

**Affiliations:** Faculty of Health Sciences, Department of Physical Therapy, Ariel University, Ariel, Israel

**Keywords:** Diagnostic markers, Musculoskeletal system

## Abstract

Backward walking offers a unique challenge to balance and ambulation. This study investigated the characteristics of spatiotemporal gait factors and ankle kinematics during backward walking in people with chronic ankle instability. Sixteen subjects with chronic ankle instability and 16 able-bodied controls walked on a treadmill at their self-selected speed under backward and forward walking conditions. Gait speed, cadence, double limb support percentage, stride time variability, and three-dimensional ankle kinematics were compared between groups and conditions. During backward walking, both groups had significantly slower gait speed, lower cadence, and greater stride time variability. In addition, under backward walking condition, subjects in both groups demonstrated significant sagittal and frontal kinematic alternations, such as greater dorsiflexion and inversion following initial contact (0–27.7%, 0–25.0% of gait cycle respectively, p < 0.001). However, there were no significant differences between groups in any of the measured outcomes. This indicates that subjects with chronic ankle instability adapt to self-selected speed backward walking similarly to healthy controls. Assessments with more challenging tasks, such as backward walking with dual task and backward walking at fast speed, may be more appropriate for testing gait impairments related to chronic ankle instability.

## Introduction

Chronic ankle instability (CAI) may be present in up to 40% of individuals who have previously experienced lateral ankle sprain^1^. CAI is characterized by repetitive episodes and subjective feeling of ankle ‘giving way’, and symptoms such as pain, swelling and limited motion^2–4^. Compared to healthy controls, individuals with CAI report quality-of-life deficits and functional limitations in addition to the physical impairments^2,5,6^.

While mechanical factors, such as ankle ligaments hyperlaxity, may be responsible for CAI in some patients^2^, it can occur even when the mechanical constraints at the ankle are intact^7^. Recent evidence suggest that CAI can be explained by sensorimotor deficits^3,7,8^. Arthrogenic neuromuscular inhibition^9,10^, ankle muscle weakness^4,11^, reduced ankle range of motion^3,9^, impaired sense of joint position^12^, and postural control are found in CAI.

Altered movement patterns during functional tasks, including walking, are often described in individuals with CAI^13^. During walking, subjects with CAI may exhibit typical kinematic patterns of increased ankle inversion and a laterally deviated center of pressure throughout the stance phase of gait^6^. Conversely, Chinn et al.^5^ reported that CAI subjects demonstrated more inversion while jogging but not while walking.

Linear variability measures that investigated the amplitude of variability, such as coefficient of variation, and non-linear variability approaches that evaluated the dynamic aspects of variability using mathematical tools related to chaos theory, have both reported differences in gait variability between individuals with and without CAI. For example, individuals with CAI have increased variability in location of center of pressure during the initial stages of stance phase^14^. In addition, differences were also found in the coordinated movement of segments of the lower extremity. For example, during walking, patients with CAI demonstrated less variability in frontal-plane ankle-hip coupling and greater variability in ankle frontal-knee sagittal-plane motions than patients without CAI^15^. Challenging conditions, such as rapid movements, fast walking, and walking with a cognitive dual task, emphasize differences in movement patterns between subjects with CAI and healthy individuals^8,16^. This indicates that higher attentional demands during a functional task may further limit the ability of this population to control movement.

Backward walking (BW) is an activity with additional complexity compared to regular forward walking (FW). Although some studies have shown that BW is a mirror image of FW, considering that initial contact during BW is done by the toe instead of the heel^17,18^. BW differs from FW in many aspects. Step length is shorter and gait speed is slightly slower during BW^19^. Kinematic and kinetic studies demonstrated greater range of ankle dorsiflexion, reduced plantar flexion, and more even plantar pressure distribution ^20,21^. BW requires greater muscle activity and has higher metabolic costs^18,19,22^. Moreover, BW involves increased activation of the sensorimotor control system due to altered or absent visual feedback^18,19,23^. BW may be extremely novel even for healthy individuals. Kurz et al.^24^ investigated gait variability and cortical activation in healthy adults during FW and BW and reported increase in sensorimotor cortical activation measured by functional near infrared spectroscopy and a greater stride-time variability during BW. It has also been reported that exercise of BW in untrained healthy adults caused neural adaptations^25^. Overall, BW offer a unique challenge to balance and movement. Thus, BW is used increasingly in rehabilitation programs to promote balance control^17^.

Due to the nature of a relatively untrained and challenging gait task, assessing characteristics of BW among people with CAI, as compared to healthy controls, may provide additional information regarding sensorimotor control in this population. Furthermore, the ability to walk backward may be a useful measure of mobility, as well as balance training strategy. Thus, it is important to quantify the performance of BW in people with CAI.

Therefore, this study investigated the characteristics of spatiotemporal gait factors and ankle kinematics during BW among people with CAI. For this purpose, we compared changes between FW and BW among individuals with and without CAI. We expected that only BW would differ between these populations. Specifically, we hypothesized that during BW, individuals with CAI would have slower gait speed and increased ankle inversion, as compared to healthy controls.

## Methods

### Participants

The sample size for this study was determined based on a power analysis calculation that was conducted using G*Power version 3.1^26^. To detect a true and meaningful difference of 0.1 m/s in BW gait speed, with standard deviation of ± 0.1, power of 80%, and a 95% confidence level, a sample of 16 subjects in each group was needed. Consequently, 16 subjects with CAI and 16 healthy controls participated in the study. The enrollment criteria for the CAI group were based on previously established standards to identify individuals with CAI^2,27^. Participants with CAI were included if they met the following criteria: (i) history of at least one significant ankle sprain that occurred at least 12 months prior to the study and was diagnosed by a physician or a physical therapist based on clinical examination^28^, (ii) history of at least two episodes of ‘giving way’ (regular occurrence of uncontrolled and unpredictable episodes of excessive inversion of the rear foot) and feelings of ankle joint instability, (iii) the most recent injury occurred more than 6 weeks prior to study enrollment, (iv) answering “yes” to at least five yes/no questions of the Ankle Instability Instrument developed by Docherty et al.^29^. This should include the first question: “Have you ever sprained your ankle?” and at least four other questions related to the severity of ankle symptoms, and (v) able to bear full weight on the injured lower extremity with no more than mild discomfort. The control group included healthy participants with no history of ankle sprain. Exclusion criteria for all groups were a history of ankle fracture, other pathological conditions or surgical procedures in the lower extremity and vestibular or neurological disorders. Participants were recruited from a university setting and provided written informed consent prior to participating in the study. Ariel University Institutional Review Board approved the study.

### Procedure

The study was conducted during one visit at the Neuromuscular and Human Performance Laboratory, at Ariel University. Gait was evaluated under both FW and BW walking conditions, while participants walked on a treadmill (VO2 Challenger, Taiwan). Participants were given standard instructions to walk at their comfortable, self-selected pace. Before data collection, subjects were provided with an opportunity to habituate to walking on the treadmill. They walked barefoot and wore tight, black sports pants and t-shirts. To capture gait data, markers were placed directly on the skin using double-sided tape. A total of 15 reflective markers were placed on each side of the participant’s iliac crest, anterior superior iliac spine, posterior superior iliac spine, greater trochanter, lateral and medial femoral condyles, tibial tuberosity, ankle medial malleolus, ankle lateral malleolus, heel, first toe metatarsal head, first toe metatarsal base, fifth toe metatarsal head, fifth toe metatarsal base, and second and third metatarsal base. In addition, cluster markers were placed at mid-thigh and mid-calf. A six-camera motion capture system (Qualisys, Göteborg, Sweden) sampled at 250 Hz was used to obtain three-dimensional ankle kinematics and the spatiotemporal data. Data was exported to Visual 3-D software (C-motion, Inc., Kingston, ON, Canada), and processed through a 6-degree of freedom anthropometric model. Ankle angles during walking were calculated using the cardan rotation sequence^30^. To normalize the gait cycle, gait events were identified automatically, as suggested by Zeni et al.^31^ and De Asha et al.^32^.

Under each walking condition, 17 consecutive strides were recorded for each participant. Then, the first and last strides were omitted, and the remaining 15 strides were analyzed. In the CAI group, the tested limb was the involved limb. The limb used for analysis in the control group was matched to the CAI by side (right or left). The spatiotemporal outcomes examined were gait speed (m/sec), cadence (steps/min), the percent of the gait cycle spent in double limb support (%DLS), and stride time variability (100 × [standard deviation of stride time/mean stride time]). Outcomes of ankle kinematics included the average and 95% confidence interval (CI) of sagittal and frontal ankle angle throughout the gait cycle.

### Statistical analysis

Descriptive statistics included mean and standard deviations (SD). Normal distribution of continuous data was verified using Shapiro–Wilk test. Simple chi-square and t-tests were used to compare baseline characteristics between the CAI and control groups. A two-way linear mixed model was performed for each spatiotemporal gait outcome with the factors of group (CAI, healthy control) and walking condition (FW, BW). The interaction effect was evaluated to determine if there were differences between groups in their walking adaptation from forward to backward. To analyze the kinematic parameters, mean sagittal and frontal ankle angles were plotted throughout the gait cycle with their corresponding 95% CI, as was previously described^5,14^. A significant difference was defined in case non-overlapping CI was found. In addition, a two-way repeated measures ANOVA using Statistical Parametric Mapping (SPM) was used to analyze the effects of group, condition and interaction (group x condition) of the kinematic data. Significance was determined as P < 0.05. The analysis was conducted using IBM SPSS, v24.0 (SPSS, Armonk, NY: IBM Corp) and the SPM1D v.0.4 package for Python 3.7^33^.

## Results

### Subject characteristics

Subject characteristics are summarized in Table 1. There were no differences in baseline characteristics (age, height, weight and sex) between groups. The average time since last sprain in the CAI group was 20.5 (18.18) weeks and the average Ankle Instability Instrument score was 6.00 (1.15).

**Table 1:** Subject characteristics.

Parameter	CAI	Control	P value
Age (years)	25.44 (2.39)	25.56 (3.44)	0.57
Height (m)	1.71 (0.11)	1.72 (0.10)	0.82
Weight (kg)	71.69 (13.82)	68.36 (12.44)	0.40
Sex (F/M)	8F/8M	9F/7M	0.72
Ankle with recurrent sprains (RT/LT)	14/2	–	–
Time since last sprain (weeks)	20.5 (18.18)	–	–
Ankle instability instrument score	6 (1.15)	–	–

*CAI* chronic ankle instability, *RT* right, *LT* left.

### Spatiotemporal gait outcomes

**Figure 1:**
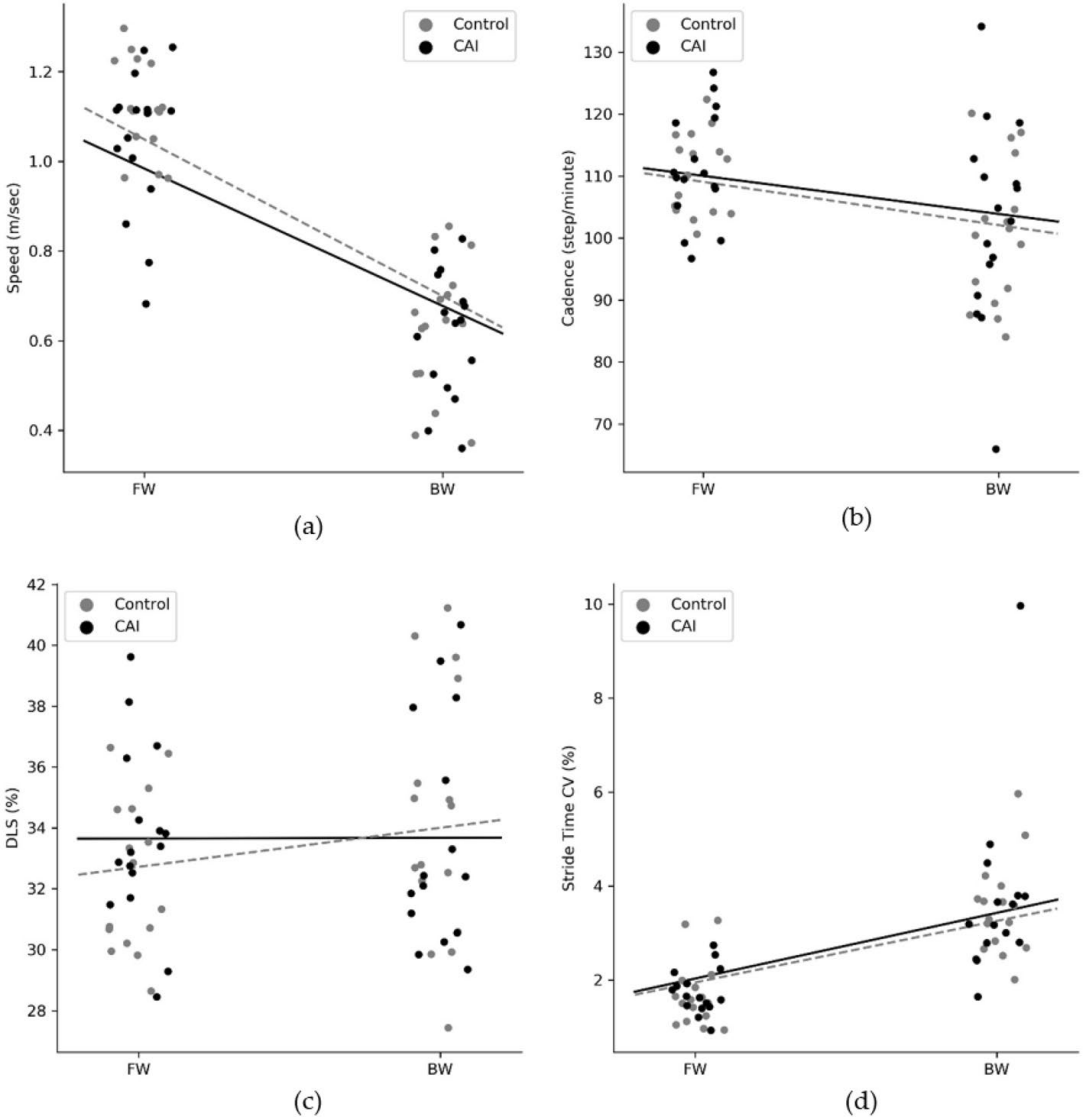
(**a**–**d**) Spatiotemporal gait characteristics during forward and backward walking. *CAI* chronic ankle instability, *DLS* double limb support, *FW* forward walking, *BW* backward walking.

The changes in spatiotemporal characteristics from FW to BW in both groups for all four spatiotemporal gait outcomes, and the results of the linear mixed model are presented in Table 2 and Fig. 1a–d.

**Table 2:** Means and (standard deviations) of spatiotemporal outcomes under each walking condition in both groups, and the linear mixed model results.

Spatiotemporal gait outcomes	CAI		Control		Estimated fixed effects		
	FW	BW	FW	BW	Group	Condition	Interaction
Speed (m/s)	1.05 (0.16)	0.62 (0.14)	1.12 (0.10)	0.63 (0.15)	P = 0.318	P < 0.001	P = 0.277
Cadence	111.25 (8.89)	102.65 (15.93)	110.45 (6.53)	100.69 (11.51)	P = 0.671	P < 0.001	P = 0.806
DLS (%)	33.65 (2.94)	33.68 (3.74)	32.46 (2.45)	34.26 (4.07)	P = 0.676	P = 0.120	P = 0.260
STV (%)	1.75 (0.48)	3.71 (1.92)	1.68 (0.69)	3.52 (0.99)	P = 0.687	P < 0.001	P = 0.826

*CAI* chronic ankle instability, *DLS* double limb support, *STV* stride time variability, *FW* forward walking, *BW* backward walking.

The analysis showed a significant effect of condition for gait speed, cadence, and stride time variability. During BW, both groups had slower gait speed (p < 0.001), lower cadence (p < 0.001), and higher stride time variability (p < 0.001). However, there was no significant group or interaction effects for these parameters, indicating no difference between groups in their adaptation to BW. In addition, no between-condition difference was evident in %DLS, as well as no between-group difference in both conditions (FW and BW).

### Ankle kinematics

**Figure 2:**
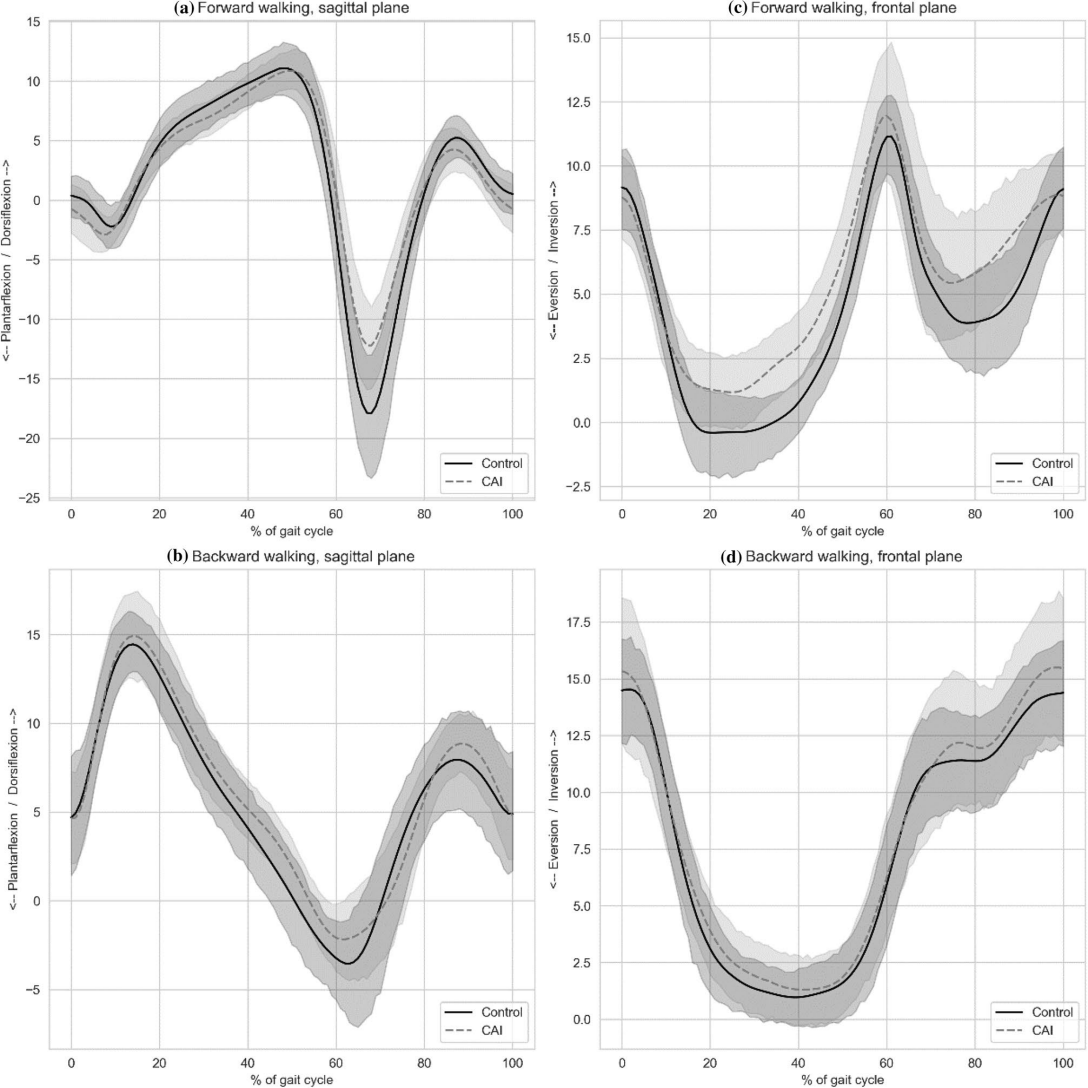
(**a**–**d**) Between-group sagittal and frontal ankle kinematic comparisons during forward and backward walking. *CAI* chronic ankle instability, *FW* forward walking, *BW* backward walking.

**Figure 3:**
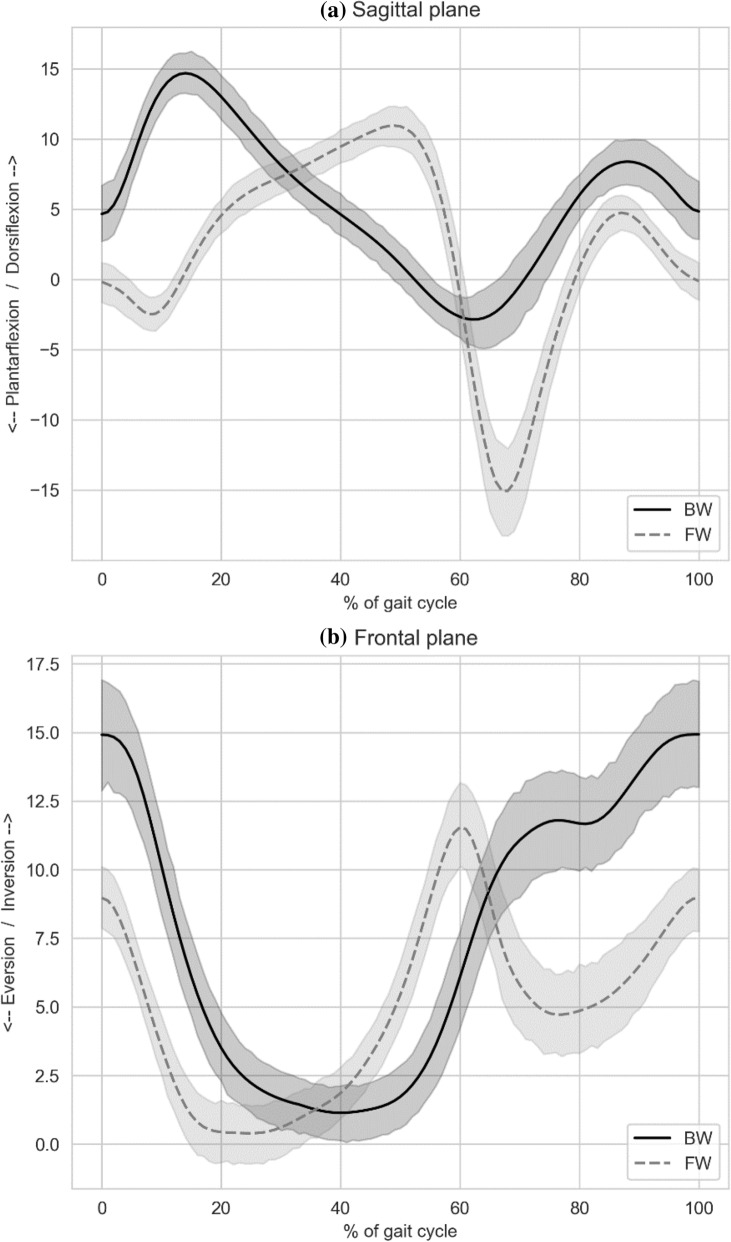
(**a**, **b**) Between-conditions sagittal and frontal ankle kinematic comparisons (including the entire sample). *FW* forward walking, *BW* backward walking.

Figure 2 presents mean sagittal and frontal plane ankle kinematics with their corresponding 95% CI under both walking conditions. As depicted in the Fig. 2, overlap of ankle kinematics CIs between the CAI and healthy controls were consistent throughout the gait cycle, indicating no significant difference between groups. Similarly, Fig. 3 presents between conditions (i.e. FW vs. BW) comparison of ankle kinematics, demonstrating no consisted CIs overlapping, indicating significant differences. The SPM analysis indicated that significant between-condition differences were found both in the sagittal and frontal plane. In the sagittal plane, BW demonstrated greater dorsiflexion at 0–27.7% of the gait cycle (p < 0.001), greater plantarflexion at 34.5–58.9% of the gait cycle (p < 0.001), and greater dorsiflexion at 62.4–100.0% of the gait cycle (p < 0.001). In the frontal plane, BW demonstrated greater inversion at 0–25.0% of the gait cycle (p < 0.001), greater eversion at 46.0–62.0% of the gait cycle (p < 0.001), and greater inversion at 67.8–100.0% of the gait cycle (p < 0.001). No significant group × condition interaction was found.

## Discussion

The current study found that under BW condition, spatiotemporal and ankle kinematics gait characteristics of subjects with CAI and healthy controls were significantly different when compared to FW. For example, during BW, gait speed was reduced, whereas stride time variability and ankle dorsiflexion were increased. Yet, while major differences were found between BW and FW, there were no differences between groups. These indicate that during BW, subjects with CAI adjust their spatiotemporal and ankle kinematics characteristics similar to the way healthy controls do. To the best of our knowledge, this study is the first to document these results.

While several studies have clearly demonstrated substantial differences in movement analysis between subjects with and without CAI^5,6,13,13^, our findings are consistent with previous reports that did not find differences between individuals of these two groups. Two recent reviews indicated that postural stability assessments using a stable surface with the eyes open may not always discriminate between individuals with CAI and healthy controls^4,34^. De Noronha and colleagues^35^ reported no differences in proprioception or motor control between CAI and a control group. A systematic review with meta-analysis conducted to determine the ability of functional performance tests to differentiate between individuals with CAI and healthy controls, concluded that clinical implementation of these tests should be limited, due to inconsistent results^36^.

Furthermore, one of the most common characteristics that has been reported in the literature that differs between patients with CAI and healthy participants is greater inversion of the foot relative to the tibia during walking^6^. Yet, conflicting results were reported in other studies where CAI subjects were not found to have more inversion^5,37^ or even have greater rearfoot eversion^38^. Similarly, the results of the present study did not observe increased ankle inversion in the CAI group in FW or BW, as compared to controls.

Several explanations may be suggested for the inconsistencies observed between studies. It is possible that some discrepancies were due to the heterogeneity of the CAI population. Hertel and Corbett ^39^ recently presented an updated model of CAI. According to this model, there is a list of impairments that people with CAI as a group are likely to demonstrate; however, each individual may present certain clinical and performance outcomes that are affected by personal and environmental factors. It seems that the inconsistencies between studies may be partially explained by this model.

Specifically, all the CAI participants in the current study met established standards for CAI ^27^. However, according to the Ankle Instability Instrument, only 3 of 16 participants reported that they feel unstable while walking on a flat surface. Previous studies have shown that only very complex walking situations, such as walking with a cognitive dual task, may differentiate CAI subjects from controls ^8,16^. Thus, it is possible that while BW on a treadmill required some level of adaptation from both groups, it was not challenging enough to discriminate between their gait performance. Assessments with more challenging tasks, such BW with dual task and BW at fast speed may be more appropriate for testing gait impairments related to CAI.

Another aspect that may have affected the results is related to the procedure of data collection. In the present study, data were collected while subjects were barefoot, as this state detects frontal plane kinematics more accurately. Systematic reviews indicated significant differences in kinematics, kinetics and muscle activity during barefoot and shod walking and running^40,41^. Likewise, previous research with CAI participants has shown that gait outcomes vary when data are collected during barefoot walking^42–44^ or with shoes^45,46^. For example, Herb et al.^45^ evaluated gait kinematics while the subjects wore shoes and reported on differences in shank-rearfoot coupling between CAI and control groups across gait cycle. The authors explained the results by altered sensorimotor function in the CAI group due to their ankle pathology. In contrast, our results did not demonstrate differences in movement analysis between subjects with and without CAI, even under a task that requires greater sensorimotor activation such as BW. A possible explanation for this difference may be related to the uniqueness of barefoot walking. Barefoot walking promotes higher plantar loading, resulting with enhanced afferent feedback of proprioception, which is desirable for control of gait and kinematic adjustments. Furthermore, BW walking relies more on proprioception rather than on visual feedback. Thus, the augmented feedback provided by the barefoot walking may increase the ability of the sensorimotor system to organize movement patterns.

Although BW did not distinguish between groups, it affected the spatiotemporal and kinematic variables in both groups, compared to FW. This finding is in agreement with previous studies that reported changes in spatiotemporal and kinematic characteristics in young adults during BW, as compared to FW^19,20,23,47^. Consistent with previous research, BW was characterized by slower gait velocity, reduced cadence, increased ankle dorsiflexion and decreased plantar flexion^20^. The present study also documented increased stride time variability, which may indicate less stability during BW^48^.

Until recently, BW was considered to be a simple reversal of FW. It was hypothesized that a single spinal mechanism controls both FW and BW^49,50^. However, current evidence suggests that BW utilizes additional elements, presumably supraspinal, in addition to a common spinal drive^18,22^. The significant adaptations during BW in both groups, and particularly the increased stride time variability, may support the notion that control of BW mechanisms may require more central nervous system resources than does FW.

Another interesting finding of the current study is related to sagittal ankle kinematics. Subjects with CAI were reported to have decreased peak ankle dorsiflexion during FW walking compared to healthy controls^5^. In the current study, the dorsiflexion peak ankle during FW was similar to findings of previous studies with CAI^5,43^ with no difference between groups, but greater dorsiflexion (+ 4.31°) was observed under the BW condition. Emerging research suggests that BW can improve locomotion in patients with neurological lesions, as well in patients with musculoskeletal disorders. A recently published study reported the effectiveness of BW as a rehabilitation technique for patients after anterior cruciate ligament reconstruction^51^. Based on our results, clinicians may consider training subjects with CAI under varied BW conditions in order to enhance their sensorimotor control of ambulation. Furthermore, the increased ankle dorsiflexion during BW may suggest that this condition can be utilized to gain greater ankle dorsiflexion. To the best of our knowledge, there is no published data to document the effectiveness of BW training for patients with CAI. Thus, future research should be performed to confirm the effectiveness of this intervention.

In the present study, gait was evaluated while the subjects walked on a treadmill. When walking over-ground, a constant speed is not usually sustained for a long period of time^52^. In contrast, during treadmill walking, the speed is fixed. The constant speed and rhythm during treadmill walking may influence the spatiotemporal and kinematics variables. However, while changes in spatiotemporal variables and hip and knee kinematics were demonstrated, ankle kinematics seem to be similar under forward self-selected walking over ground and on a treadmill^53–55^. This may support the ecological validity of the findings regarding ankle kinematics during FW in the current study.

As far as we know there are no studies that compared BW over a treadmill to BW over ground. Visual information is important for maintaining equilibrium and stability during locomotion. While walking backward visual information is limited and the subject cannot observe potential obstacles. Furthermore, during over-ground locomotion the subject moves with respect to the surroundings, while during treadmill walking the opposite occurs, and the surroundings moves with respect to the subject. This may add more complexity to visual perception during BW treadmill walking. Thus, future studies that will compare BW over ground and on a treadmill are warranted.

This study had several limitations. It was originally powered to identify differences between the groups for gait speed and did not account for the additional spatiotemporal and kinematic variables. Additional limitations are that we did not separate CAI subjects according to mechanical and functional instabilities, and the subjective report of perceived instability during BW was not tested. Updated models of CAI indicate differences between mechanical and functional instability among individuals with CAI and stresses the importance of evaluating self-reported perceived instability^39^. Thus, further investigations with a larger cohort should be undertaken to confirm the study results and assess and analyze relevant subgroups of patients with CAI.

## Conclusions

Participants with CAI and healthy controls demonstrated significant changes in spatiotemporal and ankle kinematics gait characteristics between BW and FW conditions. However, there were no significant between-group differences in both conditions, indicating that subjects with CAI adjust their spatiotemporal and ankle kinematics characteristics during BW similar to the way healthy controls do. Clinicians should consider this information with caution when assessing and designing training programs for individuals with CAI, due to the heterogeneity of this population.

## Data Availability

The datasets used and analyzed during the current study are available from the corresponding author on reasonable request.
